# Early Detection and Identification of *Methylobacterium radiotolerans* Bacteremia in an Early T-Cell Precursor Acute Lymphoblastic Leukemia Patient: A Rare Infection and Literature Review

**DOI:** 10.3390/pathogens14101015

**Published:** 2025-10-07

**Authors:** Jiayu Xiao, Lingli Liu, Xuzhen Qin, Yingchun Xu

**Affiliations:** 1 Department of Clinical Laboratory, State Key Laboratory of Complex Severe and Rare Diseases, Peking Union Medical College Hospital, Peking Union Medical College, Chinese Academy of Medical Sciences, Beijing 100730, China; xiaojiayukang@163.com (J.X.); liulingli@163.com (L.L.); 2 Graduate School, Peking Union Medical College, Chinese Academy of Medical Sciences, Beijing 100730, China

**Keywords:** *M. radiotolerans*, blood culture system, Myco/F Lytic culture vial, antibiotic susceptibility testing

## Abstract

(1) Background: *Methylobacterium radiotolerans* (*M. radiotolerans*) is a fastidious, aerobic, Gram-negative bacillus primarily found in environmental sources such as soil and sewage, with rare clinical isolation. Its identification remains challenging due to poor growth with conventional culture methods. (2) Case presentation: A 42-year-old male patient with early T-cell precursor acute lymphoblastic leukemia (ETP-ALL) presented with *M. radiotolerans* bacteremia during hospitalization. The organism was successfully isolated from peripheral blood using the Myco/F Lytic culture vial (Becton, Dickinson and Company, Lincoln, MT, USA). Comparative analysis demonstrated markedly superior growth of *M. radiotolerans* in Myco/F Lytic culture vials compared with Plus Aerobic/F Lytic and Lytic/10 Anaerobic/F culture vials (Becton, Dickinson and Company, Lincoln, MT, USA). Antimicrobial susceptibility testing, performed with the epsilometer test (E-test) and Bauer–Kirby disk diffusion (BK) method, guided the selection of an appropriate therapeutic regimen. The patient’s infection was ultimately controlled following targeted antimicrobial therapy. (3) Conclusions: *M. radiotolerans* demonstrates a distinct growth preference for the Myco/F Lytic culture medium. This observation highlights the importance of considering alternative culture media in cases of rare or fastidious bacterial infections that cannot be reliably detected using conventional Plus Aerobic/F Lytic or Lytic/10 Anaerobic/F culture vials, which are typically employed for clinical isolation of aerobic and anaerobic bacteria.

## 1. Introduction

*M. radiotolerans* is an aerobic, slow-growing, Gram-negative bacillus that forms characteristic pink-pigmented colonies [1]. This bacterium is predominantly isolated from environmental sources such as leaf surfaces, soil, and sewage [2,3]. Although generally considered to have low pathogenicity, it acts as an opportunistic pathogen in immunocompromised individuals, particularly those with underlying conditions such as leukemia, end-stage renal disease, or organ transplantation [4,5]. Of note, indwelling intravascular devices represent a major risk factor for bloodstream infections, and most of the previously reported clinical cases have been catheter-related [5,6]. Furthermore, as a pseudomonad, *M. radiotolerans* is capable of forming surface-associated capsules and demonstrates tolerance to chlorine-based disinfectants and elevated temperatures [7]. These traits facilitate its persistence in healthcare environments, where it has been detected in hospital tap water [3], creating opportunities for nosocomial transmission.

Notably, due to its fastidious nature, *M. radiotolerans* is hardly detectable under conventional culture conditions, which may partly explain the limited number of documented clinical infections [4,8,9,10]. Here, we report a confirmed case of *M. radiotolerans* bloodstream infection identified via the BACTEC™ Myco/F Lytic Culture Vial (Becton, Dickinson and Company, Lincoln, MT, USA), describe its morphological features during culture, and present its antimicrobial susceptibility profile.

## 2. Case Description

**Figure 1:**
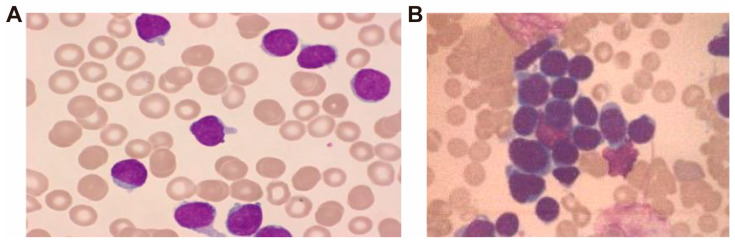
Cytomorphological features of peripheral blood (**A**) and bone marrow (**B**) smear, Wright-Giemsa stain, ×400.

On 2 July 2024, a 42-year-old male with ETP-ALL was admitted with a 10-day history of sore throat, enlarged submandibular lymph nodes, and intermittent fever. On admission, vital signs were stable, and physical examination revealed a 2 × 2 cm ulcerated, crusted wound on the right ankle. Peripheral blood smear revealed numerous round or oval blasts, characterized by large nuclei with finely granular chromatin, well-defined nuclear membranes, occasional nuclear indentations or clefts, and one to two prominent nucleoli. The cytoplasm was minimal, exhibiting a pale blue hue (Figure 1A). Bone marrow smear demonstrated markedly hypercellular proliferation, predominantly composed of pro-lymphoblasts. The blasts exhibited large nuclei with finely dispersed chromatin, thickened nuclear membranes, occasional nuclear indentations and clefts, and prominent nucleoli, typically single. The cytoplasm ranged from scant to moderate in volume, with a gray-blue appearance, occasionally containing small vacuoles (Figure 1B). Specific laboratory tests are presented in Table 1.

For ongoing management, a peripherally inserted central catheter (PICC) was placed on 5 July. On hospital day 6, following initiation of chemotherapy, the patient developed a fever (38.6 °C). Peripheral blood cultures were obtained using BACTEC™ Lytic/10 Anaerobic/F and Plus Aerobic/F Lytic Culture Vials, but no organisms were isolated. The fever resolved transiently with meropenem and loxoprofen. However, on 11 July, the patient experienced recurrent fever (38.3 °C). Repeated blood cultures using the same culture vials remained negative. The patient still had recurrent fever despite empirical treatment with vancomycin and loxoprofen. Multiple subsequent cultures over the following days yielded no growth after 7 days of incubation.

On 21 July 2024, the patient developed a high-grade fever (40.4 °C) accompanied by a marked elevation in PCT (33 ng/mL). Empiric antimicrobial therapy was escalated to include vancomycin, imipenem, and caspofungin, alongside antipyretics including intravenous lysergic acid, indomethacin suppositories, and oral loxoprofen. Concurrently, peripheral blood was inoculated into Plus Aerobic/F Lytic, Lytic/10 Anaerobic/F, and Myco/F Lytic culture vials. Only the Myco/F Lytic vial flagged positive at 75 h; the other vials remained negative after 7 days.

**Figure 2:**
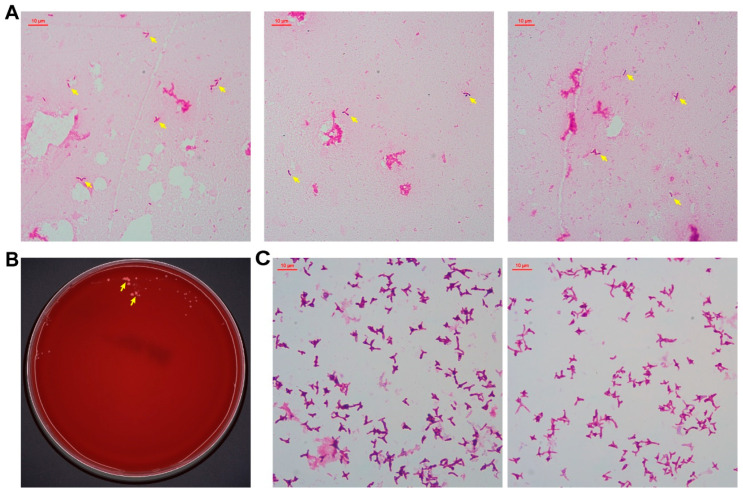
Cell morphology and colony appearance of *M. radiotolerans* isolates. Gram staining after growing in Myco/F Lytic culture vials for 75 h, and these arrows point to *M. radiotolerans* (**A**). After 4 days on blood agar, and these arrows point to *M. radiotolerans* colonies (**B**). Gram staining after 5 days on blood agar (**C**).

Gram staining of the positive culture revealed rod-shaped and occasionally bifurcated Gram-negative bacilli (Figure 2A). Subculture on blood agar and China blue agar plates (37 °C, 5% CO_2_) yielded sparse, small pink colonies on the blood agar plate after four days (Figure 2B). Identification via matrix-assisted laser desorption/ionization time-of-flight mass spectrometry (MALDI-TOF MS) (ZHUHAI DL BIOTECH Co., Ltd., Zhuhai, China) confirmed *M. radiotolerans* with >99.9% confidence, which was corroborated by 16S rRNA gene sequencing (GenBank accession: PV362230; performed by Beijing Ruibo Xingke Biotechnology Co., Ltd., Beijing, China).

**Figure 3:**
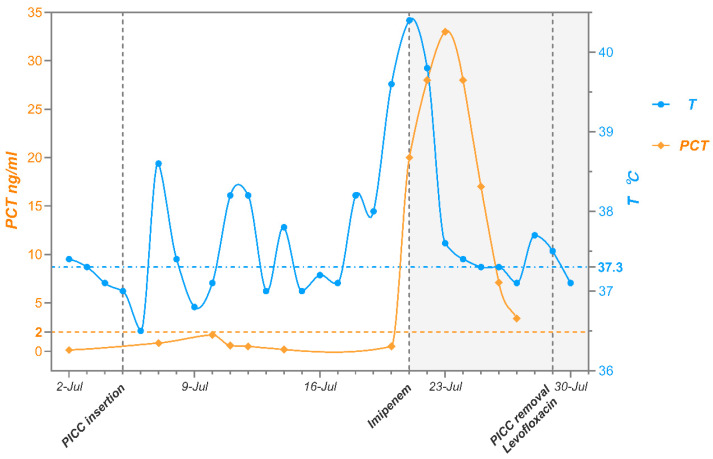
Temperature and PCT trends during the patient’s hospitalization.

Following laboratory identification of the pathogen, the PICC was removed, and antimicrobial therapy was adjusted to imipenem combined with levofloxacin based on experience from previous *M. radiotolerans* infection case reports [6]. The patient subsequently experienced defervescence and a gradual decline in PCT levels (Figure 3). The patient was discharged on 31 July 2024, with a one-week course of oral levofloxacin. At follow-up two weeks later, the patient remained afebrile and free of infectious symptoms.

## 3. Additional Microbiological Studies

In this case, the isolation and identification of *M. radiotolerans* were pivotal for diagnosis and therapeutic decision-making. Notably, the organism was successfully detected only in the Myco/F Lytic culture vial, while multiple cultures using Lytic/10 Anaerobic/F and Plus Aerobic/F Lytic culture vials remained negative. This observation suggested that *M. radiotolerans* may have a growth preference for the Myco/F Lytic culture medium.

To evaluate this hypothesis, a controlled experiment was conducted. A standardized suspension of *M. radiotolerans* (10–50 CFU/mL) in sterile saline was inoculated (1 mL per vial) into Lytic/10 Anaerobic/F, Plus Aerobic/F Lytic, and Myco/F Lytic culture vials [11]. All vials were incubated in the BD BACTEC™ FX Blood Culture System (Becton, Dickinson and Company, Baltimore, MD, USA) for up to 14 days. Only the Myco/F Lytic culture vials flagged positive, with an average detection time of 71 h; the other vials remained negative. Subsequent subculture on Mueller-Hinton agar confirmed the presence of *M. radiotolerans*, supporting the feasibility of Myco/F Lytic culture vials for cultivating this fastidious organism.

Prior to strain identification, routine clinical cultures using blood agar and China blue agar incubated at 37 °C in 5% CO_2_ yielded limited growth, with only small colonies observed on the blood agar plate, consistent with previous reports [5]. Given the slow growth rate of *M. radiotolerans*, antimicrobial susceptibility testing (AST) using the VITEK 2 Automated System (bioMérieux, Craponne, France) was not feasible. Therefore, AST was conducted using standard methods for Gram-negative bacilli, including the E-test and BK methods.

**Figure 4:**
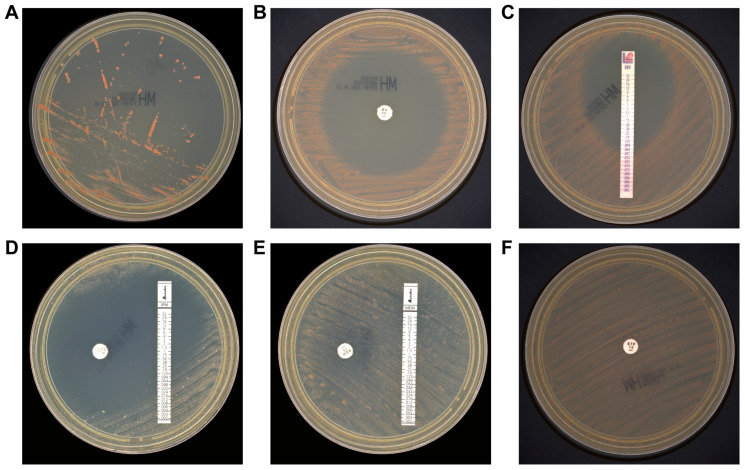
Colony morphology and antibiotic susceptibility of *M. radiotolerans* isolates. Colonies appeared on Mueller-Hinton agar after 3 days (**A**). AST on Mueller-Hinton agar for 5 days. Minocycline (**B**), eravacycline (**C**), imipenem (**D**), meropenem (**E**), and ertapenem (**F**).

Considering earlier studies reporting poor growth of *M. radiotolerans* on Mueller-Hinton (MH) and blood MH agar under standard conditions [5], we subcultured the isolate onto MH agar and incubated it at 30 °C. After three days, satisfactory growth was observed (Figure 4A). Following five days of incubation, clear zones of inhibition were noted around several antibiotic discs (Figure 4B–F). To rule out contamination, multiple colonies with varying morphology from the AST plates were randomly selected for MALDI-TOF MS identification, all confirming *M. radiotolerans* with high confidence (>99.9%). All agar media used in this study were produced by Thermo Fisher Scientific Biochemical Products (Beijing, China) Co., Ltd.

AST results are presented in Table 2. Neither EUCAST nor CLSI has established specific clinical breakpoints for *M. radiotolerans* [12]. Therefore, antimicrobial susceptibility interpretation was extrapolated from general susceptibility patterns. Consistent with previous reporting practices, and considering that *M. radiotolerans* is taxonomically classified within the *Pseudomonas* genus, we referred to the interpretive criteria established for standard antimicrobial susceptibility testing of *Pseudomonas aeruginosa* [8,9]. Nevertheless, owing to the prolonged incubation period required for *M. radiotolerans* isolates, a more conservative approach was applied when evaluating antibiotic susceptibility. In terms of carbapenems, the isolate exhibited susceptibility exclusively to imipenem, while showing resistance to meropenem and ertapenem. It also demonstrated susceptibility to tetracyclines, gentamicin, and levofloxacin, but was resistant to cephalosporins, vancomycin, and aztreonam, findings that are largely consistent with previous reports [3,8].

## 4. Discussion and Conclusions

*M. radiotolerans* is an opportunistic environmental bacterium, with only a few clinical cases reported to date [9,13]. This may be attributed to its low pathogenicity or the challenges associated with its isolation and identification in clinical settings. Table 3 presents the clinical features of *M. radiotolerans* infections documented in the literature to date. These infections typically occur in immunocompromised individuals with severe underlying conditions. Patients often exhibit pronounced symptoms and limited response to commonly used empirical antibiotics [8]. Delayed or missed diagnosis in such cases can lead to severe consequences [9], underscoring the critical importance of accurate and timely identification.

Among these infected patients, leukemia is the most common underlying condition. Similar to the patient in this case, leukemia patients experience severe disruption of the survival space for normal hematopoietic stem cells due to their bone marrow being occupied by malignantly proliferating leukemia cells. This leads to a sharp decline in the number of key immune cells such as neutrophils and lymphocytes. Furthermore, leukemic cells themselves exhibit functional abnormalities, rendering them incapable of mounting effective immune responses [16]. Simultaneously, they secrete multiple immunosuppressive factors (such as TGF-β and IL-10), creating an inhibitory microenvironment that further weakens the function of remaining normal immune cells [17]. Moreover, treatments like chemotherapy or radiotherapy, while eliminating cancer cells, also inflict further damage on the already compromised immune system, resulting in persistent and severe immune deficiency, such as treatment-related neutropenia [18]. This creates an opportunity for pathogens with low pathogenicity like *M. radiotolerans* to thrive.

This article reports for the first time that *M. radiotolerans* bacteremia can be effectively detected using Myco/F Lytic blood culture vials, ensuring timely smear microscopy and subculture of positive samples. Final identification was achieved through MALDI-TOF MS or 16S rRNA sequencing. This approach offers a practical workflow for clinical microbiology laboratories, contributes to strain database enrichment, and supports the refinement of diagnostic and therapeutic strategies for bloodstream infections. In cases of suspected septic shock with persistently negative results from standard Plus Aerobic/F Lytic and Lytic/10 Anaerobic/F culture vials, Myco/F Lytic culture vials may enhance the detection of this fastidious organism, thereby guiding targeted antimicrobial therapy and potentially improving outcomes in rare bacterial infections.

This study has several limitations. First, the mechanism underlying the improved recovery of *M. radiotolerans* using Myco/F Lytic culture vials, compared with Plus Aerobic/F Lytic and Lytic/10 Anaerobic/F culture vials, remains unclear. One possible explanation is that *M. radiotolerans* may be better adapted to low-nutrient environments, whereas nutrient-rich media could suppress its growth [6]. As a fastidious bacterium with a preference for carbon–carbon bond–containing compounds [19], previous reports have shown that it grows well on Sabouraud agar but poorly on chocolate and blood agar [5,8], suggesting that medium composition may directly influence its proliferation. Alternatively, specific components within the Myco/F Lytic vials may promote its growth. Further studies are required to clarify these possibilities and to establish optimal culture conditions that will enhance the isolation and identification of this organism. Second, in vitro antimicrobial susceptibility testing revealed an unusual carbapenem resistance pattern: *M. radiotolerans* was highly susceptible to imipenem but exhibited complete resistance to meropenem and ertapenem. The molecular mechanisms underlying this discrepancy remain unknown and warrant further investigation.

Unexplained fever is a thorny problem frequently encountered in clinical practice. This case emphasized the significance of sample collection and submission methods for the detection rate of fastidious bacteria. The successful detection via Myco/F Lytic culture vials underscores their potential utility in improving early diagnosis of this rare organism and may provide a valuable approach for cases with negative standard culture results. 

## Figures and Tables

**Table 1:** Specific laboratory tests during hospitalization.

Category	Time Period			Reference Values
	Pre-Hospitalization	Intra-Hospitalization	Post-Hospitalization	
RBC, ×10^12^/L	3.44	1.56	2.38	4.00–5.50
HB, g/L	106	50	73	120–160
WBC, ×10^9^/L	98.03	0.28	5.70	3.50–9.50
N (%)	0	14.3	79.6	50.0–75.0
LYM (%)	6	82.1	7.9	20.0–40.0
Blast (%)	94	-	-	-
PLT, ×10^9^/L	36	10	105	100–350
LDH, U/L	467	90	139	0–250
hsCRP, mg/L	29.70	311.65	36.19	<3.0
Bone marrow flow cytometry	Abundant early abnormal T lymphocytes.	-	-	-
Bone marrow smear	Hypercellular proliferation, predominantly composed of prolymphoblasts (96.5%).	-	Adequate cellularity with granulocytic predominance (85%) and a relative increase in segmented neutrophils.	-

Abbreviations: RBC, red blood cell counts; HB, hemoglobin; WBC, white blood cell counts; N%, percentage of neutrophils; LYM%, percentage of lymphocytes; PLT, platelet counts; LDH, lactate dehydrogenase; hsCRP, high-sensitivity C-reactive protein.

**Table 2:** Antimicrobial susceptibility testing results for *M. radiotolerans*.

Class	Antibiotic	Inhibition Zone Diameter (Disc-Diffusion) (mm)	MIC (mg/mL)
Carbapenem	Imipenem	40	0.75
	Meropenem	6	>=32
	Ertapenem	6	-
Aminoglycosides	Gentamicin	31	-
	Amikacin	-	1.5
4-quinolones	Levofloxacin	27	0.5
	Ciprofloxacin	-	2
Tetracyclines	Minocycline	43	-
	Tigecycline	26	-
	Eravacycline	-	0.125
Cephalosporins	Ceftriaxone	-	3
	Cefoxitin	6	-
Glycopeptides	Vancomycin	6	-
Monobactams	Aztreonam	6	-

**Table 3:** Clinical features of *M. radiotolerans* infections are described.

Year, [Reference]	Underlying Disease(s)	Catheter-Related	Source(s) of Isolated Bacteria	Identification Method(s)	Antibiotic Susceptibility	Therapy	Outcome
2009 [4]	Acute/chronic renal failure	CVC	37 patients, from blood (2.7%) or CVC (29.7%) or both (67.6%)	16S rRNA sequencing	-	Removal and replacement of CVCs and antibiotic therapy.	Improved
2011 [14]	Dilated cardiomyopathy, end-stage renal disease	DLC	Blood from DLC	16S rRNA sequencing	Resistant to aztreonam, ceftazidime, cefepime, and piperacillin-tazobactam.	Removal of DLC, cefepime and ciprofloxacin	Improved
	Leukemia, febrile neutropenia	CVC	Blood from CVC			Removal of CVC, ciprofloxacin and gentamicin	
2015 [5]	AML	PICC	Blood from PICC	16S rRNA sequencing	-	Levofloxacin	Improved
	AML	PICC	Blood from PICC			Clindamycin, levofloxacin and cefepime	
	AML	PICC	Blood from PICC			Levofloxacin and linezolid	
	ALL, GVHD	CVC	Blood from CVC			Ciprofloxacin and piperacillin-tazobactam	
2015 [15]	End-stage renal failure, COPD	CVC	Blood	16S rRNA sequencing	Susceptible to amikacin, netilmicin, gentamicin, levofloxacin, piperacillin-tazobactam and meropenem.	Removal and replacement of CVC, levofloxacin and meropenem.	Improved
2019 [8]	End-stage renal failure	CVC	Peripheral blood and CVC	MALDI-TOF MS, 16S rRNA sequencing	Susceptible to gentamycin, levofloxacin, rifampicin and imipenem.	Not known	Improved
2019 [6]	Peripheral T-cell lymphoma, sick sinus syndrome	Hemodialysis vascular access	Peripheral blood and the vascular access line blood	MALDI-TOF MS, 16S rRNA sequencing	Susceptible to imipenem, amikacin, tobramycin and levofloxacin.	Removal and replacement of the vascular access line, meropenem and rifampicin for 14 days, and oral levofloxacin and rifampicin subsequently for 8 weeks.	Improved
2020 [9]	After spinal cord surgery	None	Valvar (aortic and mitral valves) vegetation tissue homogenate	MALDI-TOF MS, 16S rRNA sequencing	Susceptible to aminoglycosides and ciprofloxacin.	Tigecycline and vancomycin	Improved
2021 [10]	COPD	None	Brain lesion	MALDI-TOF MS	-	Levofloxacin	Improved

Abbreviations: CVC, central venous catheter; DLC, double-lumen catheter; AML, acute myelogenous leukemia; PICC, peripherally inserted central catheter; ALL, acute lymphoblastic leukemia; GVHD, graft-versus-host disease; COPD, chronic obstructive pulmonary disease.

## Data Availability

The dataset supporting the conclusions of this article is included in the article.

## Abbreviations

The following abbreviations are used in this manuscript:

*M. radiotolerans*	*Methylobacterium radiotolerans*
ETP-ALL	Early T-cell precursor acute lymphoblastic leukemia
E-test	Epsilometer test
BK	Bauer-Kirby disk diffusion
PCT	Procalcitonin
PICC	Peripherally inserted central catheter
MALDI-TOF MS	Matrix-assisted laser desorption/ionization time-of-flight mass spectrometry
AST	Antibiotic susceptibility testing
MH	Mueller-Hinton
CLSI	Clinical and Laboratory Standards Institute
